# 肺癌患者肺炎衣原体IgG阳性与放射性肺损伤相关细胞因子的关系

**DOI:** 10.3779/j.issn.1009-3419.2011.02.05

**Published:** 2011-02-20

**Authors:** 文熠 张, 田奎 乔, 道安 周, 素娟 袁

**Affiliations:** 1 200540 上海，复旦大学附属金山医院放射肿瘤科 Department of Radiation Oncology, Jinshan Hospital, Fudan University, Shanghai 200540, China; 2 200433 上海，同济大学上海肺科医院放射治疗科 Department of Radiation Terapy, Shanghai Pulmonary Hospital, TongJi University, Shanghai 200433, China

**Keywords:** 肺炎衣原体, 放射性肺损伤, 细胞因子, Chlamydia pneumoniae, Radiation-induced pulmonary lesion, Cytokine

## Abstract

**背景与目的:**

肺炎衣原体感染与肺癌有密切关系，但肺炎衣原体感染对肺癌放射性肺损伤的影响尚未见报道。本研究旨在探讨肺癌患者肺炎衣原体（chlamydia pneumoniae, Cpn）IgG阳性对放射性肺损伤相关细胞因子的影响。

**方法:**

观察有病理诊断、初次胸部放疗肺癌患者69例，分别于放疗前、放疗中和放疗后采血冻存，采用酶联免疫吸附法检测血液中Cpn的IgG以及IL-1β、SP-A、TGF-β、TNF-α的含量。

**结果:**

69例肺癌患者中Cpn IgG阳性21例，阴性48例，阳性率为30.43%。Cpn IgG阳性组与阴性组放疗中IL-1β浓度分别为（35.82±10.09）ng/L和（30.01±6.46）ng/L，两组之间有差异无统计学意义（*P* < 0.05），放疗前和放疗后无明显差异。Cpn IgG阳性组与阴性组放疗前SP-A的浓度差异无统计学意义，放疗中分别为（641.78±106.81）ng/L和（100.86±61.4）ng/L，有统计学差异（*P* < 0.05），放疗后浓度分别（657.47±115.19）ng/L和（93.23±47.15）ng/L，也有统计学差异（*P* < 0.05）。Cpn IgG阳性组与阴性组放疗前、放疗中、放疗后TNF-α浓度两组之间均无统计学差异。Cpn IgG阳性组放疗前、放疗中、放疗后TGF-β1的浓度分别（710.67±358.16）pg/mL、（1, 002.06±542.16）pg/mL和（2, 125.16±1, 522.29）pg/mL，阴性组分别为（867.77±412.48）pg/mL、（914.05±425.70）pg/mL和（1, 073.36±896.01）pg/mL，两组之间比较放疗后TGF-β1浓度有统计学差异（*P* < 0.05）。

**结论:**

肺癌患者Cpn IgG阳性一定程度上提高了放射性肺损伤相关细胞因子SP-A、TGF -β1、IL-1β的水平，提示可能加重肺癌患者的放射性肺损伤。

## Abstract (en)

**Background and objective:**

There exsits intimate relationship between infection with chlamydia pneumoniae (Cpn) and lung cancer incidence. But few studies have been reported about radiation-induced pulmonary lesion in lung cancer patients infected with Cpn. The aim of this study is to explore the correlation between cytokines related to radiation-induced pulmonary lesion and Cpn IgG positive in lung cancer patients.

**Methods:**

A total of 69 patients with lung cancer received chest radiotherapy. Blood samples were collected and frozen before radiotherapy (pre-RT), middle radiotherapy (mid-RT) and after radiotherapy (post-RT). Cpn IgG and levels of IL-1β, SP-A, TGF-β, and TNF-α were measured by enzymelinked immunosorbent assay (ELISA).

**Results:**

In the total of 69 patients, 21 patients were Cpn IgG positive, 48 patients negative. The positive rate was 30.43%. In mid-RT concentration of IL-1β in Cpn IgG positive and negative group were (35.82±10.09) ng/L and (30.01±6.46) ng/L, with statistically significant difference (*P* < 0.05). Pre-RT and post-RT concentrations of IL-1β in Cpn IgG positive and negative group had no statistically significant difference. Mid-RT concentrations of SP-A in Cpn IgG positive group and negative group were (641.78±106.81) ng/L and (100.86±61.4) ng/L respectively, with statistically significant difference (*P* < 0.05). Post-RT concentration of SP-A in Cpn IgG positive and negative group were (657.47±115.19) ng/L and (93.23±47.15) ng/L respectively, with statistically significant difference (*P* < 0.05). Concentrations of TNF-α in Cpn IgG positive and negative group had no statistically significant difference. Concentrations of TGF-β in Cpn IgG positive group were (710.67±358.16) pg/mL in pre-RT, (1, 002.06±542.16) pg/mL in mid-RT, (2, 125.16±1, 522.29) pg/mL in post-RT; those in negative group were (867.77±412.48) pg/mL, (914.05±425.70) pg/mL, (1, 073.36±896.01) pg/mL. Concentration of TGF-β in post-RT between Cpn IgG positive and negative group had statistically significant difference (*P* < 0.05).

**Conclusion:**

Cpn IgG positive in lung cancer patients influenced levels of IL-1β, SP-A, TGF-β during chest radiotherapy. This might aggravate radiation-induced pulmonary lesion.

放射性肺损伤是肺癌放射治疗的主要并发症，也是影响肺癌患者预后重要因素之一。近些年研究发现肺炎衣原体(chlamydia pneumoniae, Cpn)感染与肺癌密切相关^[1-8]^，但肺炎衣原体感染对肺癌放射性肺损伤的影响未见报道。本研究于2008年1月-2009年12月检测了初次接受放射治疗的肺癌患者的Cpn IgG阳性情况，并检测放射性肺损伤的相关细胞因子，初步观察肺癌患者Cpn IgG阳性与放射性肺损伤的关系。

## 1 材料与方法

### 1.1 研究对象

收集我院2008年1月-2009年12月间接受根治性放疗、有明确病理诊断的肺癌患者69例，其中男性患者57例，女性患者12例，本实验所有患者均签署知情同意书。69例肺癌患者在本实验之前未做过Cpn相关检查，无Cpn感染的急性或慢性症状及体征。所有患者分别于放疗前(开始放疗前1天)、放疗中(放射剂量达到30 Gy时)、放疗后(放射治疗结束时)采3次空腹血4 mL-5 mL，3, 000 r/min，离心5 min，分离血浆，置于-35 ℃保存待测。

### 1.2 治疗方式

6 MV直线加速器，X射线，采用常规分割方式，一天一次，一次1.8 Gy-2.0 Gy，每周5次。患者在三维TPS上进行设计计划，勾画靶区，95%的等剂量曲线包绕PTV。CTV包括原发灶(病灶外缘1 cm-2 cm)和区域淋巴结引流区，照射至40 Gy，复查胸部CT，改野或缩野。放疗总剂量50 Gy-60 Gy。肺受照20 Gy体积占全肺百分比(V20)≤20%，脊髓受照量 < 45 Gy。

### 1.3 Cpn IgG检测

Cpn IgG ELISA试剂盒购自美国R & D公司。先检测患者放射治疗前血浆中Cpn的IgG：设5个标准品孔，加标准品50 μL并梯度稀释，设2个空白孔，在样品孔先加样品稀释液40 μL，再加样品10 μL，在37 ℃水箱温育1 h，弃液，洗板5次，加酶，在37 ℃水箱温育30 min，弃液，洗板5次，先后加显色剂A和B，在37 ℃水箱温育15 min，加终止液，450 nm波长比色。

### 1.4 细胞因子检测

IL-1β、SP-A的ELISA试剂盒均购自美国R & D公司；TNFα、TGFβ的试剂盒购自美国EBIOSCINECE公司。检测方法同Cpn检测。

### 1.5 统计学分析

统计方法采用SPSS 16.0软件进行统计分析。所有计量资料组间比较采用*t*检验。计数资料分析采用卡方检验。*P* < 0.05为差异具有统计学意义。

## 2 结果

### 2.1 Cpn IgG阳性率

入组观察的肺癌患者共69例，治疗前检测Cpn IgG阳性21例，阴性48例，肺癌患者Cpn IgG阳性率为30.43%。Cpn IgG阳性组与阴性组患者的性别、年龄、吸烟史、病变分型、病理分类、临床分期、KPS等一般情况无明显差异(*P* > 0.05)(表 1)。

**1:** 临床病理参数与Cpn IgG之间的关系 The relationship between clinicopathological parameters and Cpn IgG

Clinical characteristic	Cpn IgG negative (*n*)	Cpn IgG positive (*n*)	Total	*X*^2^	*P*
Gender				0.011	0.916
Male	39	18	57		
Female	9	3	12		
Age (years)				0.05	0.824
> 60	33	15	48		
< 60	15	6	21		
Smoking status				0.007	0.936
Smoking	37	16	53		
Non-smoking	11	5	16		
Growth pattern				0.426	0.514
Peripheral type	20	7	27		
Central type	28	14	42		
Position				1.510	0.219
Left lung	26	8	34		
Right lung	22	13	35		
Pathological type				5.690	0.128
Squamous cell carcinoma	22	4	26		
Adenocarcinoma	9	5	14		
Small cell carcinoma	3	4	7		
No pathological type	14	8	22		
Stage				0.917	0.922
Ⅱa	2	0	2		
Ⅱb	4	2	6		
Ⅲa	18	8	26		
Ⅲb	24	11	35		
KPS				0.011	0.916
> 80	39	18	57		
< 80	9	3	12		

### 2.2 Cpn IgG阳性与细胞因子的含量

Cpn IgG阳性组与阴性组之间放疗前、放疗中、放疗后IL-1β的含量进行比较发现，放疗中(治疗第4周)Cpn IgG阳性组与阴性组相比IL-1β的含量差异有统计学意义(*P* < 0.05)；放疗前、放疗后IL-1β含量未见明显差异(*P* > 0.05)。放疗前Cpn IgG阳性组SP-A的含量高于阴性组，但无统计学差异(*P* > 0.05)。Cpn IgG阳性组放疗中SP-A的含量较阴性组明显增高，两组之间差异有统计学意义(*P* < 0.05)，Cpn IgG阳性组放疗后SP-A的含量升高，与阴性组之间差异有统计学意义(*P* < 0.05)。Cpn IgG阳性组与阴性组放疗前、中、后TNF-α的含量两组之间均未见明显差异(*P* > 0.05)。Cpn IgG阳性组放疗中、放疗后TGF-β1较放疗前明显升高，有明显差异(*P* < 0.05)。Cpn IgG阳性组与阴性组放疗前、治疗中TGF-β1的含量间无明显差异(*P* > 0.05)。放疗后Cpn IgG阳性组TGF-β1明显高于阴性组，有统计学差异(*P* < 0.05)(表 2)。

**2:** Cpn IgG阴性组与阳性组中的细胞因子含量（Mean±SD） Levels of cytokine in Cpn IgG negative group and Cpn IgG positive group in lung cancer patients (Mean±SD)

Cytokine	Cpn IgG negative (*n*=48)	Cpn IgG positive (*n*=21)
IL-1*β* (ng/L)		
pre-RT	34.14±12.19	35.15±11.22
mid-RT	30.01±6.46	35.82±10.09^*^
post-RT	32.23±4.61	35.42±5.64
SP-A (ng/L)		
pre-RT	80.04±44.83	346.95±81.29
mid-RT	100.86±61.4	641.78±106.81^*^
post-RT	93.23±47.15	657.47±115.19^*^
TNF-*α* (pg/mL)		
pre-RT	10.76±7.68	8.84±5.28
mid-RT	10.15±7.90	10.96±6.38
post-RT	14.41±8.25	10.73±6.04
TGF-*β*1 (pg/mL)		
pre-RT	867.77±412.48	710.67±358.16
mid-RT	914.05±425.70	1, 002.06±542.16^#^
post-RT	1, 073.36±896.01	2, 125.16±1, 522.29^#*^
#Significantly different from pre-RT in the same cytokine at *P* < 0.05; ^*^Significantly different from Cpn IgG negative at *P* < 0.05.		

## 3 讨论

肺炎衣原体是一种常见的人类呼吸道病原体，既往对于Cpn的认识往往局限于其可导致社区获得性肺炎^[9]^。近年来陆续有临床流行病学资料报道，肺炎衣原体慢性感染与肺癌的发生发展有关，值得临床重视。检索到关于肺炎衣原体感染与肺癌关系的临床流行病学的国外文献有8篇^[1-8]^，均提示Cpn慢性感染与肺癌的发生存在一定的关联。特别是3篇巢式PCR病例对照研究显示了Cpn慢性感染与肺癌发生的密切相关性。Littman等^[3]^在美国进行的调查研究设计最严谨，论证力度最强，其研究结果：随着IgA滴度的增加，OR值亦增加。对于一项样本量达508例且经过严格混杂因素控制的巢式病例对照研究而言，这一结果是可信的，从临床流行病学角度可以认为Cpn感染是肺癌的一个危险因素。Koh、Littman等^[2, 3]^的研究均显示鳞癌与Cpn慢性感染的相关性较其它病理类型更强(OR值分别为1.42和1.70)，且具有统计学意义。国内文献^[10-14]^报道肺炎衣原体慢性感染与肺癌发生有一定相关性，其中周妍等^[11]^研究Cpn与肺癌的关系，采用患者的血清及肺癌组织为材料进行研究，通过研究表明，肺癌组Cpn抗原(Cpn-Ag)表达水平(阳性率为88%)明显高于对照组，值得临床重视。Cpn的感染可导致机体产生IgG，血清Cpn抗体的检测有多种方法，如补体结合、微量免疫荧光(micro-immunofluorescence, MIF)和ELISA等方法，其中MIF和ELISA法有较高的特异性和灵敏度^[15]^。本组观察的肺癌患者共69例，肺癌患者Cpn IgG阳性率为30.43%。Cpn IgG阳性代表当前或近期曾发生感染^[16]^，而入选本实验肺癌患者均无Cpn感染的急性或慢性临床表现，提示这些患者可能为亚临床或隐性的Cpn感染。Cpn IgG阳性组与阴性组在年龄、性别、吸烟史、分型、分期、病理、KPS值等方面差异无统计学意义(*P* > 0.05)。

放射性肺损伤早期为急性炎症，逐步进展为肺纤维化，在整个过程中肿瘤坏死因子α(tumor necrosis factor-α, TNF-α)、转化生长因子β1(transforming growth factor, TGF-β1)等细胞因子与细胞及组织损伤关系密切，具有重要作用^[17]^。Cpn可被中性粒细胞或吞噬细胞吞噬杀灭，但有时不但不能杀灭，反而在细胞内繁殖，以其肺部慢性炎症。其可以刺激炎症细胞释放一系列炎症介质，如肿瘤坏死因子、白细胞介素等，以及一系列反应，从而引起肺组织的损伤。肺炎衣原体感染是否会加重放射性肺损伤，目前未见报道。

放射性肺损伤的相关细胞因子的变化早于临床改变，并且相对于临床指标，细胞因子更能客观反映情况。所以本研究通过检测几个与放射性肺损伤相关的因子，观察肺癌患者Cpn IgG阳性与放射性肺损伤的关系。

白细胞介素-1β(interleukin-1β, IL-1β)是由单核巨噬细胞产生的细胞因子，具有调节辅助T细胞活化、白细胞化学吸引、趋化粒细胞、淋巴细胞聚集、巨噬细胞刺激作用的功能。卢秀兰等^[18]^对病毒感染性肺炎患儿血浆中的IL-1β进行检测发现IL-1β在急性炎症期水平升高，而正常人血中不含IL-1β或含量很低。在本研究中，对本组69例放疗的肺癌患者血中IL-1β的含量进行检测发现，放疗前和放疗后Cpn IgG阳性组与阴性组的IL-1β的含量差异无统计学意义(*P* > 0.05)。放疗中两组的IL-1β的含量差异有统计学意义(*P* < 0.05)。提示Cpn IgG阳性患者有可能在一定程度上加重放射性肺损伤。

肺表面活性物质相关蛋白(surfactant-associated proteins, SP-A)是肺泡Ⅱ型上皮细胞和气道Clara细胞合成并分泌的亲水性蛋白。肺泡Ⅱ型细胞对放射线很敏感，肺泡Ⅱ细胞受损后可导致肺泡张力变化，肺顺应性下降，肺泡塌陷和不张^[19]^。本实验结果显示：放疗前Cpn IgG阳性组与阴性组的SP-A的含量差异无统计学意义(*P* > 0.05)。放疗中和放疗后两组的SP-A的含量差异有统计学意义(*P* < 0.05)。结果提示Cpn IgG阳性可能加重放射线对肺泡Ⅱ细胞的损伤程度。

TNF-α是由单核/巨噬细胞分泌的一种可溶性多肽，体内细胞因子调节网络的启动因子，是启动炎症反应的关键细胞因子，在放射性肺炎的发生和维持过程中占有重要地位。Jaattela^[20]^认为TNF-α能促进肺部炎症细胞的聚集，增加巨噬细胞和内皮细胞产生IL-1、TGF-β及其受体表达，刺激成纤维细胞增殖等，从而加重组织的炎症反应。臧倩等^[21]^研究认为血清TNF-α变化有可能成为放射性肺损伤的预测指标。本实验结果显示：Cpn IgG阳性组与阴性组的放疗中、放疗后TNF-α的含量较治疗前明显升高，但两组之间差异无统计学意义(*P* > 0.05)。

TGF-β1是一种多功能细胞因子，可促进胶原蛋白的分泌以及细胞外基质的沉积，是组织细胞应答放射性刺激的主要因子，其水平升高已被视为放射性肺损伤的标志之一^[22]^。本组结果显示：Cpn IgG阴性组患者放疗中、放疗后的TGF-β1含量较治疗前都有升高，但差异无统计学意义(*P* > 0.05)。Cpn IgG阳性组患者放疗后的TGF-β1含量较放疗前和放疗中也明显升高，并且差异有统计学意义(*P* < 0.05)。Cpn IgG阳性组与阴性组之间比较放疗前和放疗中的TGF-β1的含量差异无统计学意义(*P* > 0.05)。放疗后两组的TGF-β1的含量差异有统计学意义(*P* < 0.05)。结果提示，Cpn IgG阳性可能影响肺癌患者肺组织的放射性损伤程度。

综上所述，本组肺癌患者Cpn IgG阳性率达30.43%，并且一定程度上增加了放射性肺损伤相关细胞因子SP-A、TGF -β1、IL-1β的含量，提示可能加重肺癌患者的放射性肺损伤，临床方面的相关性还需要进一步深入的研究证实。
